# DCN-YOLO: A Small-Object Detection Paradigm for Remote Sensing Imagery Leveraging Dilated Convolutional Networks

**DOI:** 10.3390/s25072241

**Published:** 2025-04-02

**Authors:** Meilin Xie, Qiang Tang, Yuan Tian, Xubin Feng, Heng Shi, Wei Hao

**Affiliations:** 1 University of Chinese Academy of Sciences, Beijing 100049, China; xiemeilin6@163.com (M.X.); tangqiang22@mails.ucas.ac.cn (Q.T.); tianyuan2018@opt.ac.cn (Y.T.); fengxubin@opt.ac.cn (X.F.); shiheng@opt.ac.cn (H.S.); 2 Xi’an Institute of Optics and Precision Mechanics of CAS, Xi’an 710119, China

**Keywords:** dilated convolutional, context aggregation, YOLO, small objects’ detection

## Abstract

With the rapid development of remote sensing technology, optical remote sensing images are increasingly being used in areas such as military reconnaissance, environmental monitoring, and urban planning. Due to the small number of pixels, fuzzy features, and complex background, it is difficult for conventional convolutions to effectively extract features from small objects. To address this problem, we propose to use multi-scale dilated convolutions to increase the receptive field size of the model to adapt to changes in object size, capture multi-scale contextual information of the feature map, and extract richer object features. First, we propose a Dilated Convolutional Residual (DCR) module for high-level feature extraction in the network. Second, the context aggregation (CONTEXT) module uses remote interaction to perform associative computation on images using contextual aggregation, allowing the model to understand the global semantic information of the image. We propose a novel object detection method, DCN-YOLO, which achieves an AP50 of 56.6 on the AI-TOD dataset, effectively improving the detection accuracy and robustness of small objects in remote sensing images. It provides a new technical approach to the detection of small objects in remote sensing.

## 1. Introduction

Remote sensing images’ small-object detection refers to the task of identifying and locating small objects in high-resolution remote sensing images. This task has important application value in many fields such as military reconnaissance, environmental monitoring, urban planning, etc. Due to the characteristics of remote sensing images, such as large changes in object scale, complex backgrounds, and high similarity between objects and backgrounds, small-object detection has become a technical challenge. Traditional object detection methods rely on manual feature extraction and shallow machine learning models, which often fail to meet high accuracy and real-time requirements.

In recent years, with the rapid development of deep learning technology, deep learning-based object detection algorithms have made significant progress in the field of small-object detection in remote sensing images. These algorithms are mainly divided into two categories: one-stage and two-stage object detection algorithms. One-stage algorithms, such as the YOLO [1,2,3,4,5,6,7] series and SSD [8,9], are preferred for their fast detection speed and ease of end-to-end training; two-stage algorithms, such as Faster R-CNN [10] and Mask R-CNN [11], are slower but have an advantage in detection accuracy.

AODN [12] is used to simultaneously detect multiple object types in remotely sensed images with large scale changes. The feature extractor has been redesigned using cascaded ReLU and Inception modules, which can increase the diversity of receptive field sizes. MCGR [13] proposes a new multi-class cyclic super-resolution generative adversarial network for benchmarking object detection based on image super-resolution. The single-shot multi-box detector optimises SSD [14] to enhance the feature extraction capability of small objects in shallow networks and improve the fusion effect. The single-shot multi-box detector effectively improves remote sensing object detection in complex scenes. MSA R-CNN [15] uses an ultra-multi-scale feature extraction network to improve feature extraction from multi-scale images and is used to solve the problem of information loss in the feature pyramid network.

LSKNet [16] can dynamically adjust its larger spatial receptive field to better simulate the ranging backgrounds of various objects in remote sensing scenes. GLSANet [17] designs a global semantic information interaction module to mine and enhance the high-level semantic information in the deep feature map, thereby mitigating the obstruction of complex backgrounds to foreground objects. The feature pyramid network is optimised to improve the performance of multi-scale object detection in remote sensing images. RSADet [18] takes into account the spatial distribution, scale, and direction/shape variation in objects in remote sensing images to alleviate the problem of object occlusion and overlap. Lu proposed an end-to-end network called Attention and Feature Fusion SSD [19]. A multi-layer feature fusion structure was designed to enhance the semantic information of shallow features. A dual-path attention module was introduced to sift through feature information, suppress background noise, and highlight key features. SME-Net [20] eliminates the salient information of large objects to highlight the features of small objects in shallow feature maps and to reduce feature confusion between multi-scale objects. ABNet [21] designs an enhanced and effective channel attention mechanism to improve the feature representation capability of the backbone network, thereby reducing the obstruction of complex backgrounds to foreground objects.

DNTR [22] significantly improves small-target detection performance through an innovative noise reduction module and a transformer-enhanced detection head. However, the DNTR model has high computational requirements and low inference speed, making it difficult to achieve high-speed inference. NWD [23] effectively solves the problem of the IoU being sensitive to the position deviation of small objects by modelling the bounding box as a Gaussian distribution, which improves the accuracy of label assignment. NWD does not perform well when embedded in a target detector with NMS, and the effect has not been verified for targets that are not extremely small. RFLA [24] effectively alleviates the sample imbalance problem in small-object detection through Gaussian receptive field modelling and hierarchical label assignment. The HLA module during training requires multiple sorting and iterations, and the effect on the one-step object detector is not improved. HS-FPN [25] effectively improves small target detection performance through high-frequency feature enhancement and spatial dependency modelling and has a flexible structure and strong compatibility. However, high-frequency enhancement may introduce noise or reduce the detection accuracy of other targets due to the bias of attentional allocation to small targets. SimD [26] effectively improves the quality of label assignment for small-object detection by combining adaptive evaluation metrics for position and shape similarity and has been shown to be superior on several benchmark datasets. The fixed threshold mechanism and the lack of adaptability to mixed-size scenes are insufficient. No direct solution is proposed to address the problem of feature loss in small-object detection.

In order to deal with the small number of pixels, fuzzy features, and complex backgrounds of small remote sensing objects, we propose to use dilated convolutions to increase the receptive field size of the model, adapt to changes in object size, capture multi-scale contextual information of the feature map, and extract richer object features, thereby improving object detection performance. CONTEXT can perform correlation calculations on the whole image, allowing the model to understand the global semantic information of the image. We propose a new object detection framework, DCN-YOLO, which achieves excellent detection results on the AI-TOD dataset and provides a novel technical approach for small-object detection in remote sensing.

The main contributions of this paper can be summarised as follows:

I. The Dilated Convolutional Residual (DCR) module is proposed and applied to the high-level feature extraction process of the network. DCR can effectively expand the receptive field of the model, enabling it to flexibly adapt to dynamic changes in the object size and accurately capture multi-scale contextual information in the feature map, thereby extracting richer and more discriminative object features.

II. Context aggregation (CONTEXT) can perform correlation calculations on the whole image, allowing the model to understand the global semantic information of the image.

III. DCN-YOLO achieves an AP50 of 56.6 on the AI-TOD dataset, effectively improving the detection accuracy and robustness of small objects in remote sensing images.

## 2. Method

### 2.1. YOLOv5

The YOLOv5 model is one of the most widely used frameworks in the field of object detection and has a wide range of application scenarios in one-stage object detection. The DCN-YOLO model is based on the basic framework architecture of YOLOv5. YOLOv5 offers four model variants from small to large, namely, YOLOv5s, YOLOv5m, YOLOv5l and YOLOv5x, depending on the number of parameters and the size of the calculation. Despite the differences in model size, the basic structure remains the same. Model size is adjusted by changing the depth and width of the model, which is reflected in changes to the number of bottleneck layers and convolutional kernels. This multi-model feature gives YOLOv5 greater flexibility and versatility in practical application scenarios, allowing the appropriate model version to be flexibly selected according to different task requirements and hardware conditions. Given the comprehensive consideration of model parameters and computational complexity, YOLOv5s was selected as the baseline model in this study.

**Figure 1:**
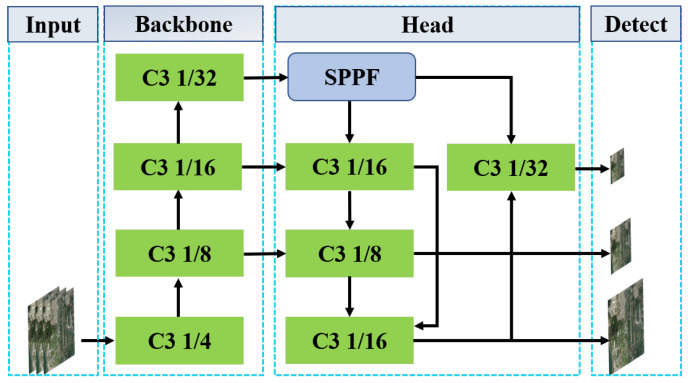
The YOLOv5 network architecture consists of three components: backbone, head, and detect.

As shown in Figure 1, the YOLOv5 network architecture consists mainly of two core components: the backbone network and the head network. The main task of the backbone network is to extract low-level texture features and high-level semantic features from the input image.

These extracted features are then passed to the head network, which achieves robust semantic feature transfer by constructing a top-down enhanced feature pyramid network while propagating local textures from bottom to top.

This bidirectional feature propagation mechanism effectively solves the problem of variable object scale by enhancing object detection capabilities at different scales, enabling the model to cope with the task of detecting objects of different sizes.

**Figure 2:**
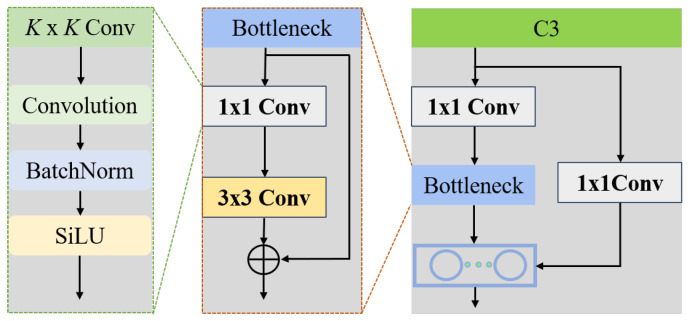
The C3 module consists mainly of bottleneck and K×KConv layers of, where *K* is the convolution kernel size.

In the backbone network and the head network, the Concentrated-Comprehensive Convolution (C3) module is a key component, as shown in Figure 2, and usually contains several bottleneck layers. These bottleneck layers consist of two successive convolutional layers, and a residual connection is introduced between the two convolutional layers, i.e., the input is directly connected to the output. This residual connection mechanism has a significant effect in mitigating the gradient disappearance problem, which can speed up the model training process and make the model converge to an optimal parameter configuration more quickly during training.

The C3 module splits the input feature map into two parts based on the channel dimension. One part goes directly to the bottleneck layer for complex feature transformation, while the other part remains unchanged. After going through their own processing flows, the two parts of the feature map are finally merged together. This unique design strategy effectively reduces the amount of computation and improves the operational efficiency of the model without sacrificing model performance, allowing the model to maintain good performance in resource-constrained environments.

**Figure 3:**
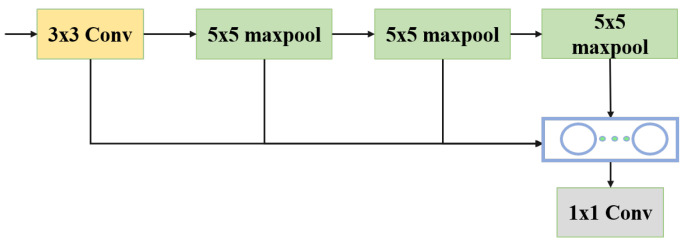
SPPF module flowchart.

The Spatial Pyramid Pooling Fast (SPPF) module is another important component of YOLOv5, as shown in Figure 3. It consists of several parallel max-pooling layers with different kernel sizes. The main function of this module is to extract deep multi-scale features from the input feature map. By extracting and fusing features at different scales, the model’s ability to detect objects of different sizes is further enhanced, allowing the model to better cope with complex and changing object scale situations.

### 2.2. DCN-YOLO

**Figure 4:**
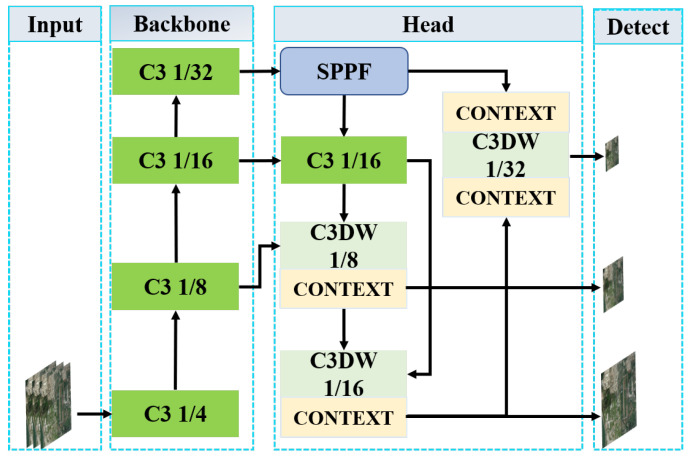
The network structure of DCN-YOLO. The DCR focuses on extracting high-level features from the network, while the CONTEXT module provides excellent long-range dependency capture, which significantly enhances the model’s ability to handle small objects.

In DCN-YOLO, as shown in Figure 4, the backbone mainly consists of the C3 module and the convolutional downsampling operation. Among them, the C3 module still plays an important role in the DCN-YOLO model to reduce the amount of computation and improve the overall performance of the network. The head part of DCN-YOLO consists of the DCR module, the Upsample layer, the Concat layer and the CONTEXT module. Among them, DCR, as an advanced convolutional residual feature extraction module, focuses on the extraction of high-level network features. This module can effectively increase the receptive field size of the model, allowing it to flexibly adapt to dynamic changes in object size, and accurately capture multi-scale contextual information in the feature map, thereby extracting richer and more discriminative object features. The CONTEXT module has excellent long-range dependency capture capabilities and can well understand the overall structure and semantic information of images, greatly enhancing the model’s processing of small objects. This allows the model to maintain high detection accuracy and stability for small objects in complex backgrounds.

Finally, the detection head part uses a combination of various loss functions such as classification loss, localisation loss, and confidence loss to achieve accurate detection of small objects in remote sensing images. The complete process of the DCN-YOLO model from feature extraction to object detection provides an efficient and accurate solution to the task of detecting small objects in remote sensing images.

### 2.3. The Dilated Convolutional Residual (DCR) Module

**Figure 5:**
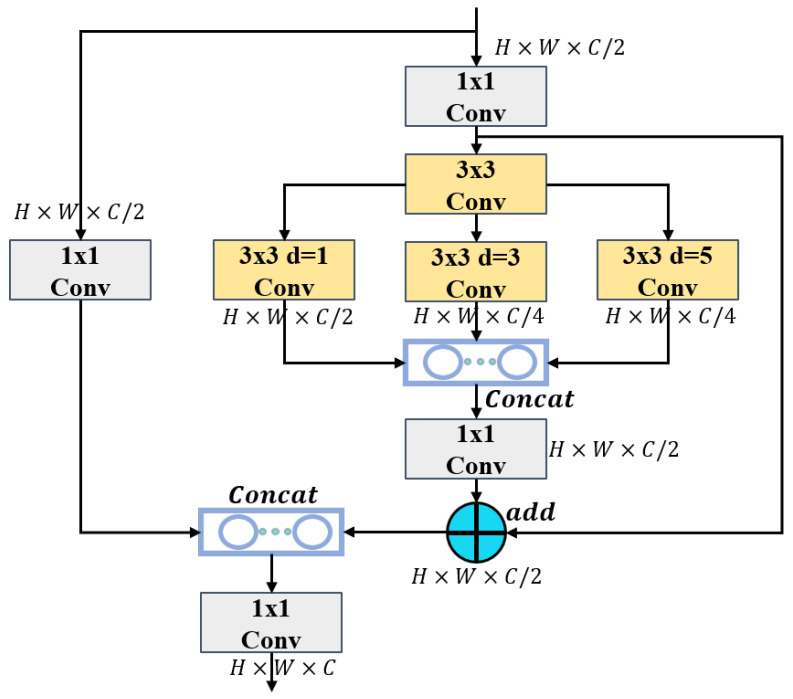
Schematic showing the DCR networking structure.

The DCR module is designed using a residual method [27], which decomposes the method of obtaining multi-scale contextual information into a two-step method, effectively reducing the difficulty of acquisition. DCR is implemented as follows: First, the size of the input feature map is $X\in R^{C\times H\times W}$ (*H*, *W*, and *C* are the height, width, and number of channels, respectively), as shown in Figure 5. The number of channels is divided into two parts. One part is subjected to a $Conv_{1,1}$ to preserve the original features of the feature map.

(1) $$Y_{1}={\mathrm{Conv}}_{1}\left(X_{C/2}\right)$$

where *Y* is the output feature map, *X* is the input feature map, and $Conv_{i,d}$ consists of the operations of convolution, batch normalisation, and the activation function SiLU, with *i* representing the kernel size of the convolution, *d* representing the dilation rate of the dilated convolution, and the other half undergoing a convolution operation with a $Conv_{1,1}$ followed by a $Conv_{3,1}$ operation, where the number of input and output channels is both C/2. A series of concise feature maps in the form of regions of different sizes are generated to provide material for morphological filtering in semantic residualisation.

(2) $$Y_{2}={\mathrm{Conv}}_{1}\left(X_{C/2}\right)$$

(3) $$Y_{3}={\mathrm{Conv}}_{3}\left(Y_{2}\right)$$

where a $Conv_{1,1}$ is used to flexibly adjust the number of channels, reducing the amount of computation while increasing the expressiveness of the features. This is followed by $Conv_{3,d}$ operations with d=1, d=3, and d=5, respectively.

(4) $$Y_{4}={\mathrm{Conv}}_{1}\left(\mathrm{Concat}\left[{\mathrm{Conv}}_{3,1}\left(Y_{3}\right),{\mathrm{Conv}}_{3,3}\left(Y_{3}\right),{\mathrm{Conv}}_{3,5}\left(Y_{3}\right)\right]\right)$$

These operations process features in different ways. Different dilation rates are applied to different groups. Depth convolution is used for morphological filtering, and only one desired receptive field is applied to each channel feature to avoid redundant receptive fields. In addition, based on the receptive field size of this step, a concise region feature map is obtained, which is required for region residual learning to reverse-match the receptive field, so that the learning process can be organised and multi-scale contextual information can be preserved more effectively. After these convolution operations, the output is fed into the Concat operation, resulting in a channel count of C/2, C/4, or C/4. The $Conv_{1,1}$ and channel Concat operations are performed again to concatenate the results with the original features of the feature map.

(5) $$Y={\mathrm{Conv}}_{1}\left(\mathrm{Concat}(\mathrm{add}\left[Y_{4}+Y_{2}\right],Y_{1}\right))$$

After the $Conv_{1,1}$ operation, the features are restored to the original H×W×C size and output.

At the same time, the DCR module adjusts the expansion rate and the capacity of the expansion convolution according to the network stage, making full use of the feature maps with different regional sizes and paying attention to the small receptive fields at each stage, expanding the capacity of the output channel of the first branch to twice that of the other branches. By repeatedly using the splicing operation, the features extracted by the previous layer can be fully reused, which improves the utilisation rate of the features and reduces the loss of information.

### 2.4. The Context Aggregation Module (CONTEXT)

The context aggregation (CONTEXT) module can implement remote interaction like a transformer [28], while taking advantage of the inductive bias of local convolution operations to achieve faster convergence. In particular, CONTEXT can perform associative computations directly on all regions of an image, allowing the model to better understand the global semantic information of the image. For regions of an image that are far apart, CONTEXT can effectively establish dependencies between them. In an image, different parts of the object may be far apart in space, but semantically related. CONTEXT can capture this long-range dependency well and thus better understand the overall structure and semantics of the image.

**Figure 6:**
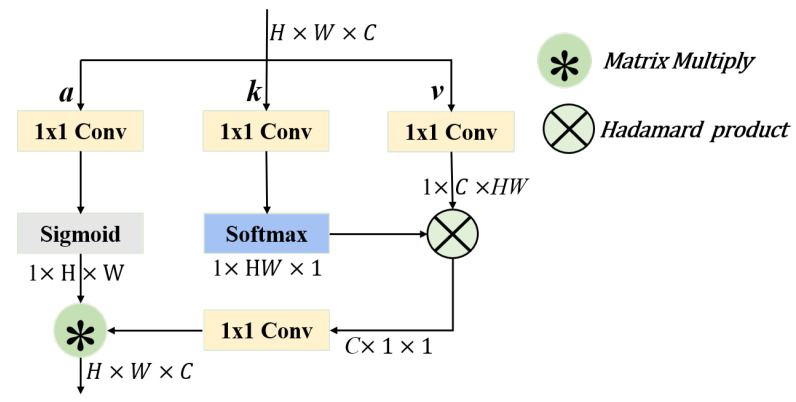
Schematic showing the CONTEXT networking structure.

Our specific implementation of CONTEXT is shown in Figure 6. The size of the input feature map is $X\in R^{C\times H\times W}$ (where *H*, *W*, and *C* are the height, width, and number of channels, respectively). The input image is first flattened into a sequence of tokens, where N=HW, and fed into the network. The network usually consists of several building blocks with remaining connections, defined as follows

(6) $$Y=F\left(X,\left\{W_{i}\right\}\right)+X.$$

where *X* and *Y* are the input and output vectors, and $W_{i}$ is the parameter to be learned. *F* determines how the information in *X* is aggregated to compute the features at a given location. First, an affinity matrix *A* is defined, which represents the neighbourhood for contextual aggregation. Equation (6) can be rewritten as

(7) $$Y=\left(AV\right)W_{1}+X,$$

where $V\in R^{N\times C}$ is the *X* transformation obtained by linear projection $V=XW_{2}$. $W_{1}$ and $W_{2}$ are trainable parameters. $A_{ij}$ is the affinity value between $X_{i}$ and $X_{j}$. By multiplying the affinity matrix by *V*, information is propagated between features based on affinity values. The modelling power of this context aggregation module can be increased by introducing multiple affinity matrices, thus giving the network multiple ways to obtain contextual information between *X*. A multiheaded version of Equation (7) is

(8) $$Y=Concat\left(A_{1}V_{1},\ldots ,A_{M}V_{M}\right)W_{2}+X.$$

Among them, $A_{m}$ represents the affinity matrix of each head. Compared to the single-head version, different $A_{m}$’s are likely to capture different relationships in feature space, thereby improving the representational ability of contextual aggregation. It should be noted that when using an affinity matrix for contextual aggregation, only spatial information is propagated; there is no cross-channel information exchange in the affinity matrix multiplication, and there is no non-linear activation function.

### 2.5. Loss Function

DCN-YOLO mainly consists of three types of loss: classification loss $\left(L_{cls}\right)$, confidence loss $\left(L_{obj}\right)$, and localisation loss $\left(L_{box}\right)$. $L_{cls}$ measures the difference between the object category in the prediction box and the real category in the real box. The Binary Cross-Entropy (BCE) loss function is used to calculate the classification error, which encourages the model to learn to correctly classify each object.

(9) $$CE_{cls}\left(p,y\right)=\left\{\begin{array}{cc} -\log(p), & \mathrm{if}\;y=1\\ -\log(1-p), & \mathrm{otherwise}. \end{array}\right.$$

(10) $$P_{t}=\left\{\begin{array}{cc} p, & \mathrm{if}\;y=1\\ 1-p, & \mathrm{otherwise} \end{array}\right.$$

(11) $$CE_{cls}\left(p,y\right)=CE_{cls}\left(p_{t}\right)=-\log\left(p_{t}\right)$$

*p* indicates the probability that the predicted sample is 1, and *y* indicates the label, which can take the values (−1, +1). For the prediction of a positive sample, the closer the predicted output is to the true sample label y=1, the smaller the loss function *L*; the closer the predicted output is to 0, the larger the *L*.

$L_{obj}$ measures the difference between the confidence in the object in the prediction box and the confidence in the object in the real box. The object confidence indicates whether the object is present in the prediction box. By optimising the loss function of the object confidence, the model can learn how to accurately determine whether the object is present in the prediction box.

(12) $$CE_{obj}\left(p,y\right)=\left\{\begin{array}{cc} -\log(p), & \mathrm{if}\;y=1\\ -\log(1-p), & \mathrm{otherwise}. \end{array}\right.$$

(13) $$P_{t}=\left\{\begin{array}{cc} p, & \mathrm{if}\;y=1\\ 1-p, & \mathrm{otherwise} \end{array}\right.$$

(14) $$CE_{obj}\left(p,y\right)=CE_{obj}\left(P_{t}\right)=-\log\left(P_{t}\right)$$

where *y* is the classification of positive and negative samples, where a positive sample is 1, and *p* is the object’s IoU score.

The $L_{box}$ measures the difference between the model’s predicted bounding box and the real bounding box, which helps the model to locate objects accurately. CIOU [29] is based on the IOU and takes into account the distance between the centroids of the real and predicted boxes, as well as the diagonal distance of the minimum enclosing box of the two boxes. When the two boxes do not overlap, the IOU is equal to 0, while CIOU can alleviate the problem of not being able to perform backpropagation because the loss is 0. CIOU is defined as follows:

(15) $$CIoU=IoU-\frac{\rho^{2}\left(b,b^{gt}\right)}{c^{2}}-\alpha v$$

(16) $$v=\frac{4}{\pi^{2}}{\left(\arctan\left(\frac{w^{gt}}{h^{gt}}\right)-\arctan\left(\frac{w}{h}\right)\right)}^{2}$$

(17) $$\alpha =\frac{v}{(1-IoU)+v}$$

(18) $$L_{box}=1-CIoU$$

where *v* measures the consistency of the aspect ratio, and α is a positive weighting parameter. The weighting parameter α is defined such that the overlap area factor has higher priority in the regression. *b* and $B_{gt}$ denote the midpoints of *b* and $B_{gt}$, $W_{gt}$ and $H_{gt}$ denote the true box’s length and width, *W* and *H* denote the predicted box’s length and width, ρ() is the Euclidean distance, and *c* is the diagonal length of the smallest enclosing frame covering both boxes.

(19) $$L=a\times L_{obj}+b\times L_{cls}+c\times L_{box}$$

where *a*, *b*, and *c* are 0.05, 0.5, and 1.0, respectively. The DCN-YOLO loss function can effectively help the model to optimise parameters and improve the accuracy and robustness of object detection.

## 3. Experiments

### 3.1. Experimental Dataset Description

#### 3.1.1. Tiny Object Detection in Aerial Images (AI-TOD) Dataset

**Figure 7:**
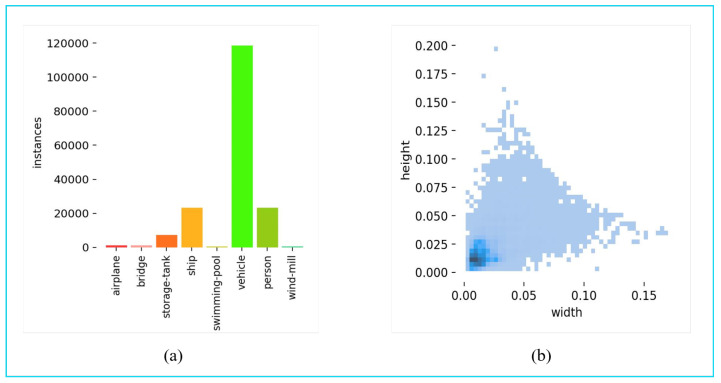
(**a**) Distribution of the number of categories in the AI-TOD dataset. (**b**) Distribution of object sizes in AI-TOD.

Tiny Object Detection in Aerial Images (AI-TOD) dataset [30] is a dataset specifically designed for the detection of very small objects in aerial images. The dataset contains 28,036 aerial images. There are 700,621 object instances in eight categories. Compared to existing aerial object detection datasets, the average size of the objects in the AI-TOD dataset is only about 12.8 pixels, which is much smaller than the object sizes in other datasets, posing a challenge to existing object detection algorithms, as shown in Figure 7. All objects are accurately labelled with bounding boxes, which is very important for model training and helps the model to accurately learn the position and shape information of the object. The images come from different geographical environments, which increases the generalisation of the model and enables the trained model to adapt to different real-world scenarios such as geographical information analysis, environmental monitoring, urban planning, and agricultural management. The AI-TOD dataset provides an important resource and benchmark for research and applications in the field of aerial micro-object detection and is of great importance for promoting the development of related technologies.

#### 3.1.2. Unicorn Small Object (USOD) Dataset

**Figure 8:**
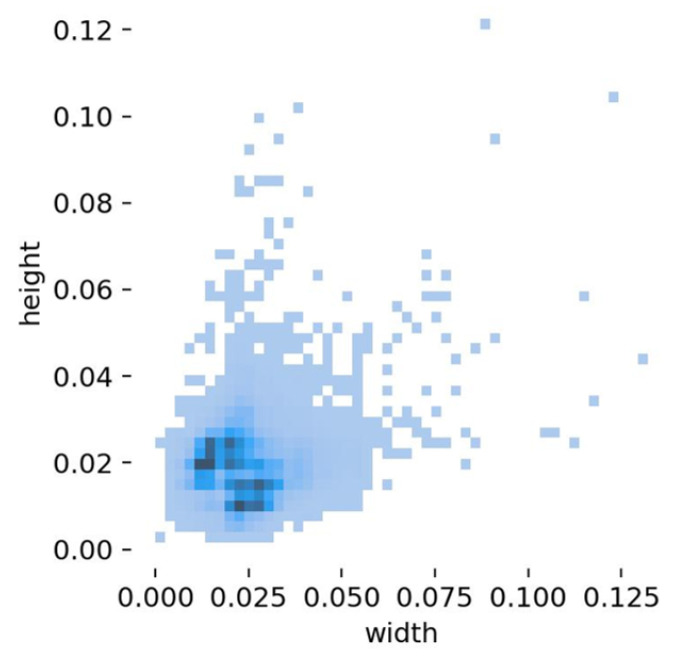
Distribution of object sizes in USOD.

The USOD was created using the visible light data from UNICORN 2008 by filtering [31], segmenting, and manually adding annotations for small vehicle objects. The USOD contains a total of 3000 images and 433,788 vehicle instances. The ratio of training set to test set is 7:3. As shown in Figure 8, the proportion of objects smaller than 16 × 16 is 96.3%, and the proportion of objects smaller than 32 × 32 is 99.9%. The proportion of small- and medium-sized objects in the USOD (99.9%) is higher than in other small object datasets. The training set has a uniform distribution of small objects. The USOD dataset contains many examples of vehicles in low light and shaded conditions, which allows the model’s performance in detecting small objects to be better verified. The USOD was used to verify the robustness of the model, taking into account image degradation factors such as blur, Gaussian noise, stripe noise, and fog.

### 3.2. Experimental Configuration and Parameter Settings

The experiment was based on an RTX3090 GPU computer, and the environment was based on Python 3.9, PyTorch 1.13, and CUDA 11.7 under the Ubuntu 18.04 operating system. The initial learning rate was set to 0.01 during training, and the minimum learning rate was 0.001. The SGD optimiser was used to update the network parameters, with a batch size of 50 and an epoch of 300.

### 3.3. Experimental Analysis

#### 3.3.1. Ablation Study

To verify the effectiveness of each key module in the DCN-YOLO model, we independently embedded the DCR and CONTEXT modules in YOLOv5 and evaluated their impact on model performance. The experimental results are presented in Table 1.

First, when the DCR module was embedded in YOLOv5, the AP value increased from 17.2 to 23.0, and the AP50 value increased from 46.0 to 49.3, indicating that the module had significant advantages in feature extraction. DCR uses multi-scale depth-wise dilated convolutions, which can effectively increase the receptive field of the model, making it more adaptable to changes in object size and thus extract richer object features. This enhanced feature expression capability further improves detection accuracy, particularly the ability to detect small objects in complex scenes.

Secondly, when the CONTEXT module was introduced alone, the AP value was improved by 4 percentage points, and the AP50 was improved by 4.8 percentage points compared to the original YOLOv5s. This result shows that the CONTEXT module enables the model to better understand the global semantic information of an image by computing global feature associations, especially when modelling the dependency between distant regions. This global perceptual capability further enhances the model’s discriminative ability and reduces the phenomenon of missed detection and misdetection of small objects due to background clutter or insufficient local features.

When the DCR module was used in conjunction with the CONTEXT module, the performance of the model was further improved, with the AP50 value increasing significantly to 56.6 and the AP value increasing to 23.9. This result shows that the DCR module and the CONTEXT module complement each other well in feature extraction and global information modelling. The DCR module improves the ability to extract local features, allowing the model to capture more fine-grained object information, while the CONTEXT module improves the efficiency of using global information, making the detection results more robust. The combination of the two effectively improves the model’s ability to detect small objects, further reducing the rate of misses and false positives, and showing significant advantages in the AP50 metric.

Overall, the experimental results show that the introduction of the DCR and CONTEXT modules play a key role in improving the overall performance of DCN-YOLO. Their synergy significantly enhances the model’s performance in the small-object detection task, providing an optimal solution for high-precision object detection.

#### 3.3.2. Comparative Analysis by Categories

As shown in Table 2, the performance advantages and potential shortcomings of DCN-YOLO were analysed in detail by comparing the performance of different algorithms in each category. DCN-YOLO achieved high detection accuracy in several categories, reaching an accuracy of 27.2 in the aircraft category, higher than YOLOv6’s 11.7 and YOLOR’s 11.7; an accuracy of 40.9 in the storage tank category, higher than YOLOv7’s 36.7 and YOLOR’s 29.0; and an accuracy of 41.9 in the ship category, higher than YOLOv6’s 38.7 and YOLOv7’s 33.7. This demonstrates the effectiveness and generalisability of DCN-YOLO in detecting small objects of different types, and its ability to adapt to the characteristics of small objects in different categories to accurately identify and locate them. This shows that DCN-YOLO can effectively deal with the challenges of small object size, blurred features and complex backgrounds when processing small-object detection tasks.

However, in some categories, such as the Bridge category, DCN-YOLO’s accuracy was comparable to YOLOv7’s (22.9 vs. 22.2); in the Windmill category, DCN-YOLO’s accuracy of 3.9 was relatively low compared to some other algorithms. This may be related to the specific characteristics of the objects in these categories and the distribution and characteristics of the samples in that category in the dataset.

#### 3.3.3. DCN-YOLO Analysis on AI-TOD

**Figure 9:**
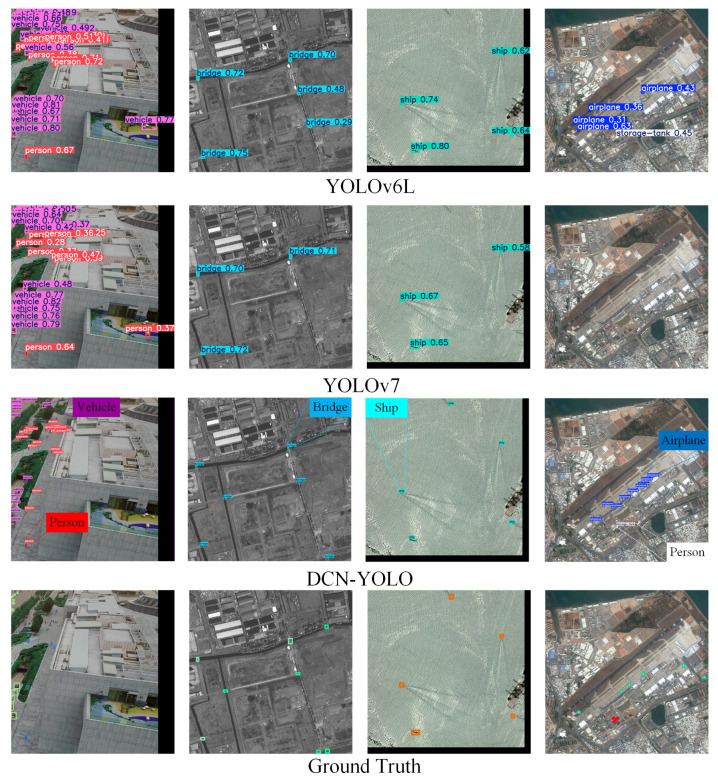
Performance of the DCN-YOLO inference on the AI-TOD dataset.

Experimental results on the AI-TOD dataset showed that DCN-YOLO achieved optimal performance in the small-object detection task, as shown in Table 3 [32,33,34,35,36,37,38,39,40,41]. It outperformed other object detection algorithms in the three core metrics, AP reaching 23.9, AP50 reaching 56.6, and AP75 reaching 15.4. DCN-YOLO’s inference performance is shown in Figure 9, demonstrating excellent detection capabilities.

DCN-YOLO achieved an AP improvement of more than 10 percentage points compared to traditional two-stage detectors (Faster R-CNN, Cascade R-CNN, and ATSS). This shows that DCN-YOLO can more effectively capture small object features and reduce missed detection by optimising feature extraction and multi-scale information fusion. At the same time, DCN-YOLO achieved an AP50 of 56.6, significantly higher than Cascade R-CNN’s 30.8 and Faster R-CNN’s 26.3, indicating its superior performance in terms of high recall detection.

Compared to other YOLO series algorithms (YOLOv5s and YOLOv8s), DCN-YOLO improved the AP metric by 6.7 and 12.3 percentage points, respectively. The DCN-YOLO had improved accuracy over the current YOLOV9 through YOLOv11 [42], indicating that it was deeply optimised for small-object detection while maintaining the high efficiency of the YOLO structure.

DCN-YOLO still showed a clear advantage over the transformer-based DETR series (DETR, Deformable-DETR and DAB-DETR). DETR performed poorly on small-object detection tasks due to the limitations of its fixed position coding, with an AP of only 2.7, while even the improved Deformable-DETR only achieved an AP of 17.0, which was still 6.9 percentage points lower than that of DCN-YOLO. This shows that DCN-YOLO avoids the inherent defects of the transformer structure in detecting small objects while maintaining high detection accuracy and has better generalisation ability and stability.

DCN-YOLO even outperformed the specially optimised small-object detection network FSANet, demonstrating DCN-YOLO’s excellent global perception capabilities.

DCN-YOLO achieved 6.4 on $AP_{vt}$, which was lower than Faster R-CNN’s 11.3, Cascade R-CNN’s 9.9, and the highest in the DETR series, Deformable-DETR’s 7.2, but higher than QueryDet’s 2.4 and FSANet’s 6.3. This shows that DCN-YOLO’s ability to detect very small objects is lower than that of the two-stage object detectors, which is attributed to DCN-YOLO’s limited feature extraction ability for extremely small targets covered by noise. For the three metrics $AP_{t}$, $AP_{s}$, and $AP_{m}$, DCN-YOLO achieved 22.1, 36.9, and 46.2, respectively. This was due to its excellent feature extraction ability in the global receptive field for targets with many pixels. DCN-YOLO has the advantages of high computational efficiency and being a lightweight model while maintaining high detection accuracy, making it more competitive in practical applications.

#### 3.3.4. DCN-YOLO Analysis on USOD

**Figure 10:**
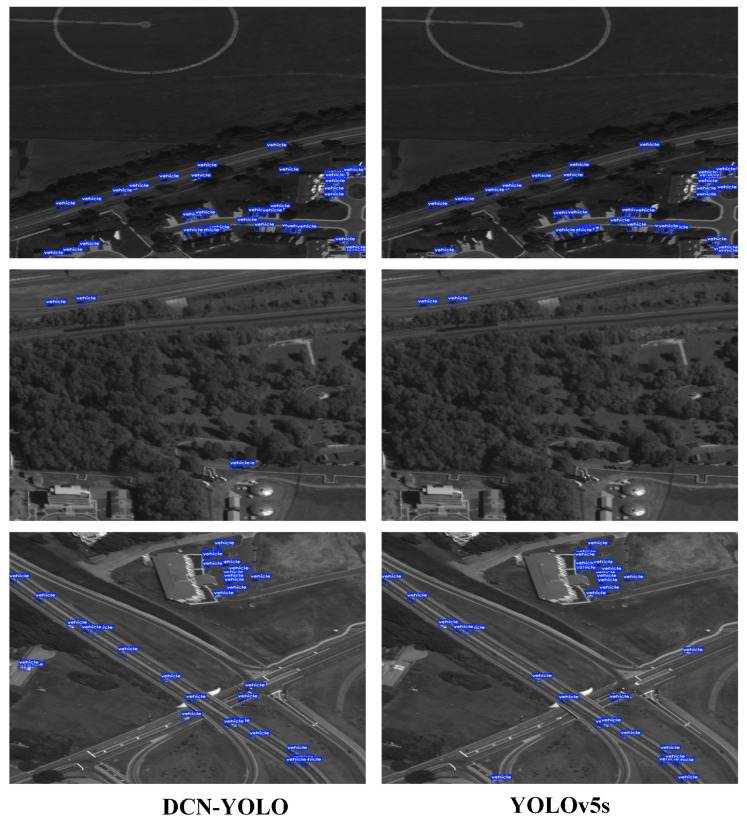
Performance of the DCN-YOLO inference on the USOD dataset.

Experimental results on the USOD dataset showed that DCN-YOLO achieved the best detection performance with extremely high parameter efficiency; the inference performance is shown in Figure 10. Compared to other mainstream object detection algorithms, DCN-YOLO performed well on key metrics such as precision (91.2), AP (48.1), and AP50 (88.5), demonstrating excellent detection capabilities and lightweight advantages, as shown in Table 4.

Compared with DSSD and RefineDet, DCN-YOLO’s precision was significantly better. At the same time, in terms of AP50, DCN-YOLO reached 88.5, which was 35.4 higher than DSSD, indicating that it had stronger detection capabilities on small objects and in complex background scenes.

Compared to YOLOv3, YOLOv4, YOLOv5m, and YOLOv8m, DCN-YOLO still maintained a leading advantage in the three core precision indicators, precision, AP50, and AP. Precision was 0.2 better than that of THP-YOLOv5 and better than that of YOLOv8m and YOLOv5m. AP50 was slightly lower than that of THP-YOLOv5, while AP reached 48.1, far exceeding all other methods, such as YOLOv8m’s 32.4 and YOLOv5m’s 32.3, an improvement of more than 15 percentage points.

In addition, DCN-YOLO had only 7.6 M parameters, making it the lightest model of all the methods. In contrast, YOLOv5m (20.9 M) and YOLOv8m (29.7 M) were 2.7 and 3.9 times larger than DCN-YOLO, respectively. THP-YOLOv5 had almost six times the parameters of DCN-YOLO, but its AP was still lower than that of DCN-YOLO (32.1 vs. 48.1).

Experimental results of DCN-YOLO on the USOD dataset show that it had the best balance between accuracy, detection performance, and model lightness. DCN-YOLO not only had the best AP and accuracy but also achieved performance far beyond other methods with a very low number of parameters (7.6 M), demonstrating extremely high computational efficiency and hardware adaptability. As a result, DCN-YOLO is currently the most advantageous object detection model for USOD tasks and is particularly suitable for application scenarios where accuracy and computational resources are tightly constrained.

## 4. Conclusions

In this study, we proposed the DCN-YOLO algorithm for the challenging task of small-object detection in remote sensing images. Through an innovative network structure design, especially the introduction of the dilated convolutional residual feature extraction module (DCR) and the context aggregation module (CONTEXT), DCN-YOLO achieved excellent performance on the AI-TOD dataset. However, there are some limitations to this research. There is room for improvement in the detection accuracy of some specific category goals, and it may be necessary to further optimise the model structure or add training strategies for specific category goals. Future work could consider further exploring how to better integrate multimodal information to further improve the model’s ability to detect small objects in complex backgrounds. At the same time, the application of the model to more practical scenarios should be explored and verified on large datasets to further verify the effectiveness and generalisability of the model. In addition, the computational efficiency and real-time nature of the model are also important considerations in practical applications. In future work, we will continue to explore more lightweight target detectors and deploy them on platforms with limited computing resources to verify performance in the real world.

## Figures and Tables

**Table 1:** Ablation study on AI-TOD testset-dev.

	AP	AP50
YOLOv5s	17.2	46.0
v5+CONTEXT	22.9	50.0
v5+DCR	23.0	49.3
DCR+CONTEXT	23.9	56.6

**Table 2:** AP of different YOLO algorithms on the single category of the AI-TOD dataset.

	Airplane	Bridge	Storage-Tank	Ship	Swimming-Pooi	Vehicle	Person	Wind-Will
YOLOv6	11.7	22	41.8	38.7	20	25.3	10.2	10.9
YOLOv7	29.9	22.2	36.7	33.7	23.3	24.8	10.5	5.1
YOLOvR	11.7	8.2	29	26.3	2.9	16.9	5.6	5.7
FCOS	0.9	17.4	29.9	43.5	4.7	24.5	4.5	1.1
Faster R-CNN	8.9	12.2	37.3	25.0	17.1	24.9	6.3	4.3
TridentNet	9.67	0.77	12.3	17.1	3.2	11.9	3.9	0.94
M-CenterNet	18.6	10.6	27.5	22.2	7.5	18.6	9.2	2.0
DCN-YOLO	27.2	22.9	40.9	41.9	25.0	26.4	11.2	3.9

**Table 3:** Test accuracy of different detectors on the AI-TOD dataset.

	AP	AP50	AP75	${\mathrm{AP}}_{\mathrm{vt}}$	${\mathrm{AP}}_{t}$	${\mathrm{AP}}_{s}$	${\mathrm{AP}}_{m}$
YOLOv9	17.9	40.7	−	−	−	−	−
YOLOv10	17.8	39.5	−	−	−	−	−
YOLOv11	17.9	41.4	−	−	−	−	−
FoveBox	8.1	19.8	5.1	0.9	5.8	12.6	15.9
DoubleHead R-CNN	10.1	24.3	6.7	0.0	7.0	20.0	30.2
Faster R-CNN	11.1	26.3	7.6	11.3	21.0	21.6	26.6
YOLOv8s	11.6	27.4	7.7	−	−	−	−
QueryDet	12.2	29.3	7.9	2.4	10.5	18.5	26.3
ATSS	12.8	30.6	8.5	4.0	14.5	21.5	31.9
Cascade R-CNN	13.8	30.8	10.5	9.9	21.3	24.1	30.3
DETR	2.7	10.3	0.7	0.7	2.1	3.0	12.4
Conditional-DETR	2.9	10.0	7.0	0.9	2.2	3.0	14.2
DAB-DETR	4.9	16.0	1.7	1.7	3.6	7.0	18.0
Deformable-DETR	17.0	45.9	8.8	7.2	17.1	22.7	28.2
DABDeformable-DETR	16.5	42.6	9.9	7.9	15.2	23.8	31.9
FSANet	20.3	48.1	14.0	6.3	19.0	26.8	36.7
YOLOv5s	17.2	46.0	−	−	−	−	−
DCN-YOLO	23.9	56.6	15.4	6.4	22.1	36.9	46.2

**Table 4:** Performance of different detectors on USOD.

	Precision	AP50	AP	Parameter
DSSD	64.5	53.1	-	-
RefineDet	88.1	85.1	-	-
YOLOv3	71.2	57.5	-	60 M
YOLOv4	79.3	77.8	-	64 M
YOLOv5m	89.2	87.3	32.3	20.9 M
YOLOv8m	90.5	87.6	32.4	29.7 M
TPH-YOLOv5	91.0	89.5	32.1	45.4 M
DCN-YOLO	91.2	88.5	48.1	7.6 M

## Data Availability

The data and code can be obtained from the corresponding author.
